# Life-threatening pulmonary embolism seen as hyperdense clots on unenhanced CT

**DOI:** 10.36416/1806-3756/e20240311

**Published:** 2025-01-10

**Authors:** Ana Paula Zanardo, Rafael Domingos Grando, Rafael Ramos Rambo

**Affiliations:** 1. Programa de Pós-Graduação em Ciências Pneumológicas, Universidade Federal do Rio Grande do Sul - UFRGS - Porto Alegre (RS) Brasil.; 2. Serviço de Radiologia, Hospital Moinhos de Vento, Porto Alegre (RS) Brasil.

A 77-year-old male patient with a history of metastatic prostate adenocarcinoma (a Gleason score of 9) presented to the emergency department complaining of abdominal pain and fever. The initial examination showed no abdominal rigidity, and pulmonary auscultation was normal. However, the attending physician suspected dyspnea, despite an SpO_2_ of 96% on room air. Initial laboratory test results showed no definite signs of infection or anemia. Unenhanced abdominal and chest CT scans showed a typical postprostatectomy appearance and no acute findings in the upper abdomen or pelvis, with no consolidation or pleural effusion in the lungs. Nevertheless, hyperdense material was seen within the right and left pulmonary arteries, and the right ventricle appeared increased. The attending physician was notified, thus ensuring the clinical stability of the patient, who then underwent CT pulmonary angiography and echocardiography. A diagnosis of acute pulmonary embolism with right heart strain was established. In the context of pulmonary embolism, high attenuation in the main pulmonary arteries has shown variable and moderate sensitivity and specificity for acute pulmonary embolism in central pulmonary arteries,^1^ ^,^ ^2^ with 100% specificity in a study by Chien et al., performing better than the Wells score.^3^

[Fig f1]

**Figure 1 f1:**
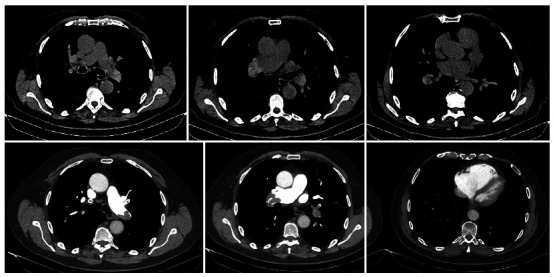
Hyperdense clots in the main pulmonary artery on unenhanced CT, confirmed by CT pulmonary angiography showing bilateral embolism and right heart strain.
